# Post-operative atrial fibrillation and stroke after non-cardiac surgery: a systematic review and meta-analysis

**DOI:** 10.1093/ehjcvp/pvaf056

**Published:** 2025-09-30

**Authors:** Jacopo Donati, Doralisa Morrone, Freek W A Verheugt, Raffaele De Caterina

**Affiliations:** University of Pisa and Cardiovascular Division, Pisa University Hospital, Via Paradisa, 2, Pisa 56124, Italy; University of Pisa and Cardiovascular Division, Pisa University Hospital, Via Paradisa, 2, Pisa 56124, Italy; Emeritus Professor of Cardiology, Amsterdam, Netherlands; University of Pisa and Cardiovascular Division, Pisa University Hospital, Via Paradisa, 2, Pisa 56124, Italy

**Keywords:** Post-operative atrial fibrillation, Non-cardiac surgery, Stroke, Risk, Rhythm monitoring, Anticoagulation

## Abstract

Post-operative atrial fibrillation (POAF) is common after non-cardiac surgery. Because often transient, there are uncertainties on the associated risk of stroke, possibly driving the need for long-term anticoagulation. We performed a systematic PubMed search until 16 January 2025, related to the incidence of stroke in patients with POAF after non-cardiac surgery. We included papers reporting outcomes, excluding studies only dealing with epidemiology, mechanisms, management, and treatment. We excluded studies reporting on POAF after cardiac surgery. Risk of bias was assessed for each study, and the certainty of evidence was evaluated using the GRADE methodology. We retrieved and included 40 studies (including review papers) for the systematic review. These were then further selected to create a final list of 19 studies included in the meta-analysis. The reported incidence of stroke after POAF was found to be widely variable, ranging between 0.4% and 16.7% at 1 year. Stroke incidence also varies widely according to the type of surgery and patient characteristics. With only three exceptions, all studies, however, reported a risk of stroke higher in the POAF group than in the no-POAF group, with a mean odds ratio of 3.02. POAF on average triples the risk of stroke, with variations related to patient characteristics and type of surgery. Patients after non-cardiac surgery should be monitored at least during hospitalisation to detect POAF. Future studies are necessary to evaluate optimal duration and modalities of monitoring, as well as to assess the relevance of symptomatic vs asymptomatic AF episodes.

## Graphical Abstract

**Graphical Abstract:**
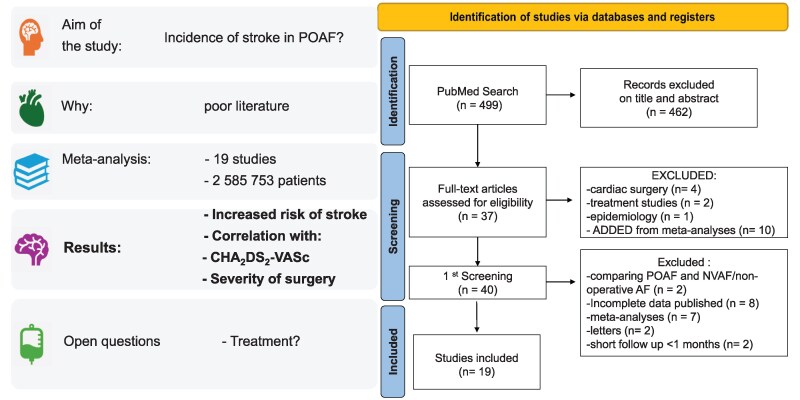
Risk of stroke in patients with post-operative atrial fibrillation (POAF) after non-cardiac surgery: search strategy and aims of the systematic review and meta-analysis, and main findings. CHA_2_DS_2_-VASc, Congestive Heart Failure, Hypertension, Age ≥75 [Doubled], Diabetes Mellitus, Prior Stroke or Transient Ischaemic Attack [Doubled], Vascular Disease, Age 65–74, Female; *n,* Number; NVAF, Non-valvular Atrial Fibrillation; POAF, Post-operative Atrial Fibrillation; PRISMA, Preferred Reporting Items for Systematic Reviews and Meta Analyses.

## Introduction

Post-operative Atrial Fibrillation (POAF) is the most common sustained arrhythmia occurring during or after surgery.^1^ Perioperative atrial fibrillation refers to AF episodes that occur in the early phases after surgery, including anaesthesia induction, intraoperative events, and the immediate post-operative period. In contrast, POAF is defined as new-onset AF occurring *after* the initial perioperative phase, typically within several days following surgery. POAF has emerged as a significant clinical complication, particularly in patients undergoing cardiac surgery.^2^ Indeed, it leads to ineffective atrial contraction and, potentially, to haemodynamic instability, extension of hospitalisation, increased healthcare costs and—most relevant—increased risk of thromboembolic events, morbidity and mortality.^3^ Pathophysiological mechanisms underlying POAF are complex and multifactorial, and include surgical stress, inflammation, fluid and electrolyte imbalances and changes in the autonomic tone. Preexisting patient characteristics, including age, history of heart disease and metabolic factors, may predispose individuals to POAF.^2^ Recognising and managing POAF is critical in current clinical practice, and involves the preoperative identification of patients at-risk, use of appropriate prophylactic measures, and institution of therapeutic interventions, with the potential of mitigating its negative prognostic impact.

POAF has been often considered a transient and therefore benign condition, and therefore mostly left untreated. Recent studies,^1,3,4^ however, have demonstrated its association with a higher risk of stroke and higher mortality compared with its absence. While many studies have explored POAF after cardiac surgery, limited data are available on its outcomes following major non-cardiac surgery, where incidence has been found to range from 0.4% to 15%, depending on the type of surgery.^2^ This figure is probably an underestimation, because only few patients after non-cardiac surgery are monitored for the occurrence of POAF. Data on POAF-related stroke risk after non-cardiac surgery remain limited in the absence of an analysis of the specific relationship between type of non-cardiac surgery and the subsequent risk of stroke.

On this background, we undertook a comprehensive systematic review of the recent literature on the risk of stroke in POAF after non-cardiac surgery to document gaps in knowledge and plan future studies on this topic, with the hypothesis that estimates on the magnitude of risk and identification of stroke predictors may inform important preventive measures.

## Methods

### Literature search and eligibility criteria

**Figure 1:**
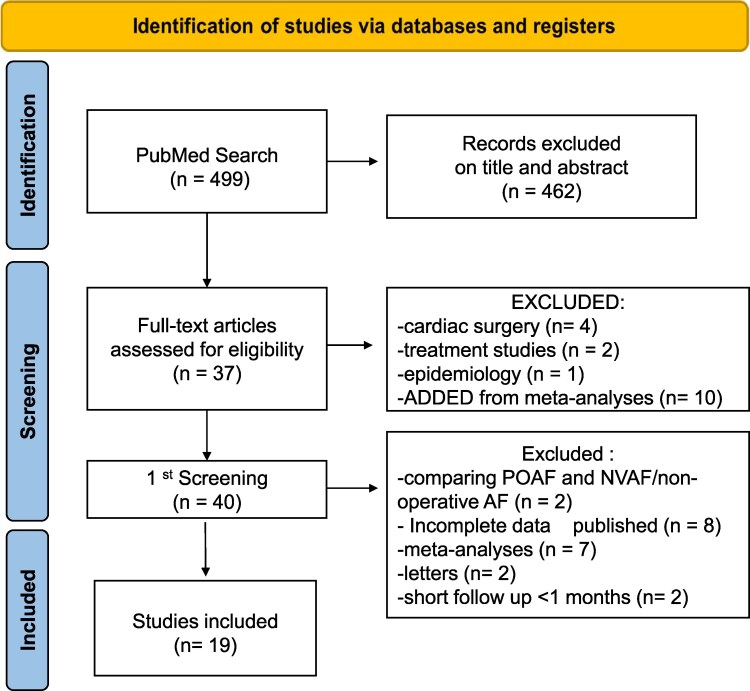
PRISMA 2020 flow chart for the present systematic review and meta-analysis. *n*, Number; NVAF, Non-valvular Atrial Fibrillation; POAF, Post-operative Atrial Fibrillation; PRISMA, Preferred Reporting Items for Systematic Reviews and Meta Analyses.

We conducted a systematic review and a study-level meta-analysis, based on the Preferred Reporting Items for Systematic Reviews and Meta-Analyses (PRISMA) 2020 statement and checklist,^5^ on the incidence of stroke in patients with POAF after non-cardiac surgery (*Figure 1*). We included articles fulfilling two criteria: (i) focusing, as main finding, on the risk of stroke; and (ii) addressing only POAF following *non-cardiac* surgery. We therefore excluded studies only reporting management, treatment, epidemiology, and mechanisms of POAF, and those on POAF after *cardiac* surgery of any kind.

The search was further restricted only to full-text articles in English, and performed through PubMed from the database inception to 16 January 2025. We used the following Medical Subject Headings terms: POAF, Post-operative atrial fibrillation, Perioperative atrial fibrillation and stroke. The research string was: (((‘POAF’) OR (‘Post-operative atrial fibrillation’)) OR (‘Perioperative atrial fibrillation’)) AND (‘stroke’). No restriction was applied as to the study type.

### Selection and data extraction

Two independent reviewers (J.D., D.M.) screened 499 articles, and disagreement on eligible studies was resolved through consensus with a senior researcher (R.D.C.). After title and abstract screening, 37 full-text articles were analyzed. We excluded 7 articles for their focusing on cardiac surgery, management, or epidemiology. An additional 10 articles were included from prior meta-analyses. Study details were listed and assessed using EndNote™ (Clarivate) and summarized in Microsoft Excel. No additional attempts were made to contact authors for missing data. Only information explicitly reported in the published articles was extracted and analyzed.

### Outcome assessment

The primary study outcome was the incidence of stroke in patients with POAF. Data on anticoagulation and antithrombotic treatments were collected when available.

### Statistical analysis

Data extraction focused on odds ratios (OR) and 95% confidence intervals (CI). A random-effects meta-analysis assessed stroke risk. Heterogeneity was evaluated using the *I*^2^ statistic. To avoid duplication of patient data, only original studies reporting individual patient-level outcomes were included in the meta-analysis, while review articles and previously published meta-analyses were excluded from the quantitative synthesis and used solely to ensure completeness of study identification. Risk of bias was assessed using a customized data extraction table based on key methodological criteria for observational studies, including population representativeness, outcome definitions, follow-up completeness, and reporting quality. Related results are presented in a dedicated table (see Supplementary material online, *Table S1*). Subgroup analyses based on clinically relevant characteristics, including surgical type (high vs intermediate-risk), mean/median age (≥70 vs <70 years), and sex distribution (≥70% vs <70% male patients with POAF), were also conducted. Also, in order to test the robustness of our findings, we performed sensitivity analyses excluding studies with unreported follow-up duration or unclear surgical classification.

## Results

### Study selection

Out of 499 studies, 40 met the inclusion criteria, with 462 articles excluded based on title and abstract, finally analyzing 37 full-text articles. We then excluded 7 of them: four^6-9^ due to the type of surgery (cardiac, valvular, CABG, thoracic aortic); two^10,11^ because only reporting management and treatment of POAF; and another^12^ because only related to epidemiology and mechanisms. Finally, we added 10 articles from previously published systematic reviews and meta-analyses. In conclusion, we included 40 articles in the present systematic review.

Out of these articles, 19 studies underwent the meta-analysis. Two studies were excluded due to reporting stroke incidence comparing patients with POAF and patients with non-valvular atrial fibrillation^4^; and patients with POAF and those with non-perioperative atrial fibrillation,^13^ whereas all the other studies reported a comparison between POAF and non-POAF patients. Seven studies^2,3,14-18^ were not part of the meta-analysis because they did not report the complete data here required. Seven other articles^19-25^ were excluded because being already meta-analyses. Two articles^26,27^ were excluded because letters in reply to an article. One study^28^ was excluded because it reported only part of the required data (452 POAF patients matched 1:1 with 452 no-POAF patients), which were the same patients reported in a second publication by the same authors.^29^ Two other articles were not part of meta-analysis because the follow-up period was <1 month.

The final analysis (*Graphical abstract*), therefore, included a total of 2 585 753 patients, of whom 29 345 with POAF and 2 556 408 without POAF. Stroke occurred in 14 204 patients, with 608 cases in the POAF group and 13 596 in the non-POAF group.

### POAF and risk of stroke

**Figure 2:**
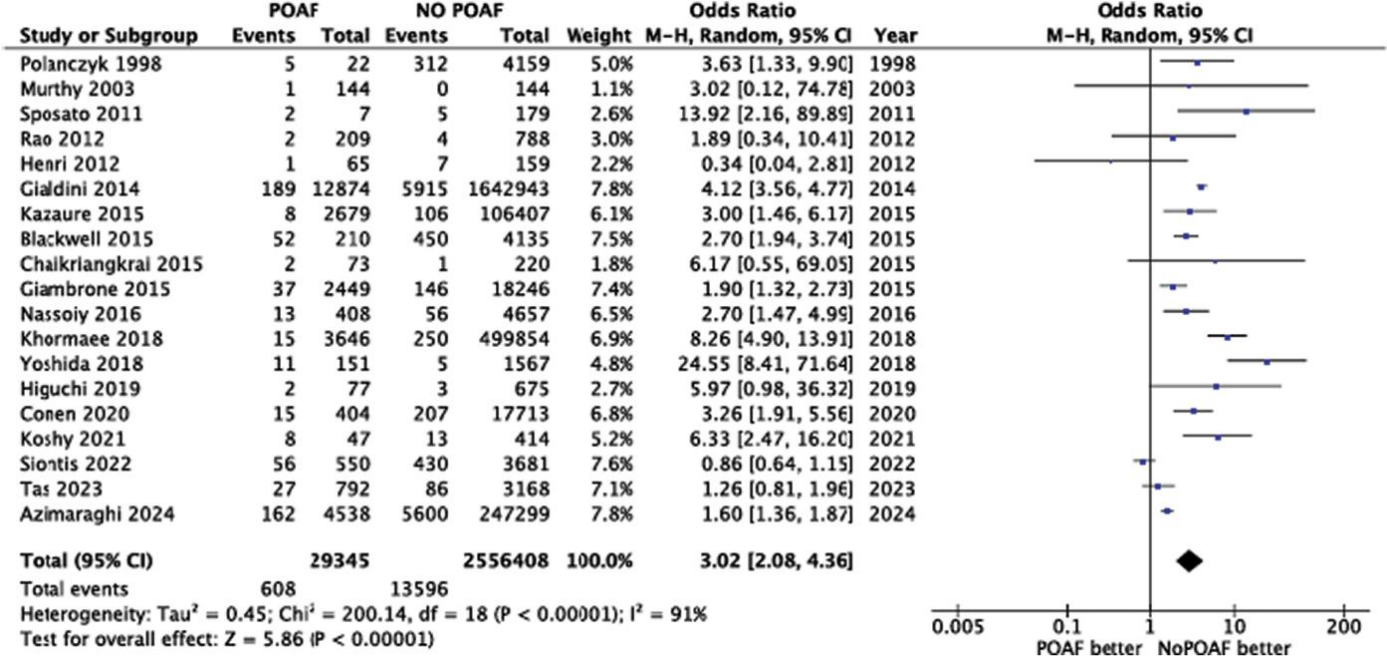
Risk of stroke after post-operative atrial fibrillation in non-cardiac surgery: forest plot of the meta-analysis. CI, Confidence Interval; df, degree of freedom; M-H, Mantel-Haenszel; POAF, Post-operative atrial fibrillation.

The pooled analysis indicated that POAF was associated with a mean three-fold increased risk of stroke (OR 3.02; 95% CI: 2.08–4.36), with significant heterogeneity among studies (*I*^2^ = 94%). The risk varied as a function of surgery type and patient characteristics. POAF appeared to be associated with a risk of stroke higher than its absence. This is demonstrated in almost all studies analyzed, with only three exceptions.^29-31^ In one study,^31^ POAF was, however, associated with a still numerically higher, although non-significantly, rate of stroke than in patients without POAF (1.5% vs 0.9% at 30 days, and 3.4% vs 2.7% at the 1-year follow-up). One study^30^ claimed that in-hospital stroke was 1.5% in POAF patients vs 1.9% in patients without POAF, in contrast with the conclusion of all the other studies and supporting the possibility that AF is a marker of comorbidities and not a major risk factor of subsequent complications. Also in the study by Siontis *et al*.^29^ the risk of stroke was found comparable between the two groups [OR 0.86; 95% CI: 0.64–1.15]. Apart from these 3 exceptions, in all other studies the risk of stroke was found higher in the POAF group than in no-POAF group, ranging from a 2.5-fold increase (RR 2.51)^32^ to a six-fold increase (OR, 6.37).^23^ The reported incidence of stroke in patients with POAF ranges, however, very widely among these studies (*Table 1* and *Figure 2*). We applied the GRADE approach to evaluate the certainty of evidence for our primary outcome, stroke (see Supplementary material online, *Table S1*). Because of the observational nature of all studies included, the starting level of certainty was considered low. The overall certainty stayed low, with no downgrading of the risk of bias (which was mostly low or moderate), indirectness (directly addressing the target population and outcome), or imprecision (OR 3.02, 95% CI: 2.08–4.36). However, the high heterogeneity across studies (*I*^2^ = 94%) led to downgrading for inconsistency. Publication bias could not be formally assessed and thus was rated as ‘undetected’.

**Table 1:** Characteristics of the studies selected

Surname of first author	First name of first author	Year of publication	Study type	Title	Brief summary related to the risk of stroke	Reference
Polanczyk	Carisi A.	1998	Prospective cohort study	Supraventricular arrhythmia in patients having non-cardiac surgery: clinical correlates and effect on length of stay	After major, non-emergency, non-cardiac procedures, 7.6% with POAF; 5 patients (total 22) with POAF with a stroke.	^33^
Amar	David	2002	Narrative review	Post-operative atrial fibrillation	330 patients undergoing NCTS; risk of stroke related to POAF 1.7% (mean follow-up 1 month).	^14^
Murthy	Sudish C.	2003	Retrospective cohort study	Atrial fibrillation after esophagectomy is a marker for post-operative morbidity and mortality	After esophagectomy, 22% with POAF; only 1 patient (total 144 patients) with POAF had a stroke.	^34^
Sposato	Luciano A.	2011	Retrospective cohort study	Intraoperative hypotension, new onset atrial fibrillation, and adverse outcome after carotid endarterectomy	Patients after carotid endarterectomy: 2 (total 7) with stroke; 5 stroke on 179 in no POAF group.	^35^
Cardillo	Giuseppe	2012	RCT	Adverse effects of fibrin sealants in thoracic surgery: the safety of a new fibrin sealant: multicentre, randomized, controlled, clinical trial	After thoracic surgery, one group with Fibrin Sealant (fibrinogen + thrombin) and the other control group. 1 POAF patient (9 total) with stroke, 1 stroke on 176 without POAF.	^36^
Henri	Christine	2012	Retrospective cohort study	Atrial fibrillation after pulmonary transplantation: incidence, impact on mortality, treatment effectiveness, and risk factors	After pulmonary transplantation, in-hospital stroke in 1.5% of POAF patients vs 1.9% without POAF; low incidence of stroke supports the conclusion that AF is a marker of comorbidities, and not a major risk factor of subsequent complications.	^30^
Rao	Vinay P.	2012	Retrospective cohort study	Age and neo-adjuvant chemotherapy increase the risk of atrial fibrillation following oesophagectomy	Patients after esophagectomy: 20.96% with POAF; in follow-up >60 months, 2 strokes on 209 POAF patients (1%).	^37^
Gialdini	Gino	2014	Retrospective cohort study	Perioperative atrial fibrillation and the long-term risk of ischaemic stroke	Considered only patients after non-cardiac surgeries (NCS); rates of ischaemic stroke in the successive one year 1.47% (POAF) and 0.36% (no POAF); risk of stroke stronger in NCS than in cardiac surgery; risk variable in proportion to CHA2DS2VASc score: 0.5% score 0, 0.7% score 1, 1.0% score 2, 1.6% score 3, 2.0% score 4, 1.9% score 5, 2.7% score6 and 2.9% score 7.	^1^
Chaikriangkrai	Kongkiat	2015	Retrospective cohort study	Incidence, Risk Factors, Prognosis, and Electrophysiological Mechanisms of Atrial Arrhythmias after Lung Transplantation	Within 30 days post-lung transplantation, 25% of patients with POAF; CHADS2 1 both in POAF and no POAF; in follow-up of 28 ± 17 months, 2/73 stroke in POAF patients.	^38^
Kazaure	Hadiza S.	2015	Retrospective cohort study	The significance of atrial fibrillation in patients aged ≥ 55 years undergoing abdominal surgery	Patients ≥ 55 years undergoing abdominal surgery: stroke in 0.3% in POAF and 0.1% in no POAF.	^39^
Blackwell	Robert H.	2015	Retrospective cohort study	Post-operative Atrial Fibrillation Predicts Long-Term Cardiovascular Events after Radical Cystectomy	Patients after radical cystectomy (for a bladder tumour); significant increase in incidence of cardiovascular accidents during first year post-operatively in POAF rather than without POAF (24.8% vs 10.9%); median time to stroke/TIA 25 days (POAF) and 21 days (no POAF).	^40^
Giambrone	Gregory P.	2016	Retrospective cohort study	Incidence and implications of post-operative supraventricular tachycardia after pulmonary lobectomy	After pulmonary lobectomy, 11.8% with POAF; stroke rate <1% in patients with POAF and 1.5% in patients with POAF with other complications; 37 on 2449 with POAF with stroke.	^41^
Nassoiy	Sean P.	2016	Retrospective cohort study	New onset post-operative atrial fibrillation predicts long-term cardiovascular events after gastrectomy	Patients undergone gastrectomy for a malignancy (pancreatic, oesophageal or gastric tumour); patients with POAF (8,1%) with higher CVA (stroke and AMI) over the 1st year after surgery than patients without POAF (16.7% vs 4.5%).	^42^
Thijs	Vincent	2017	Narrative review	Post-operative atrial fibrillation: Target for stroke prevention?	Incidence of POAF 10–20% after thoracic NCS, and 0.37–1% after NCTS; about stroke incidence, they reported Gialdini article.	^15^
Vallurupalli	Srikanth	2017	Narrative review	Controversies in post-operative atrial fibrillation after non-cardiothoracic surgery: clinical and research implications	POAF associated with increased risk of stroke at 30 days; in their opinion, short-term anticoagulation (30 days) recommended; long-term anticoagulation only with POAF >48 h and CHA2DS2-VASc was ≥2 (always talking to families before taking decision).	^16^
Ayoub	Karam	2018	Retrospective cohort study	Long-Term Risk of Recurrent Atrial Fibrillation and Ischaemic Stroke after Post-Operative Atrial Fibrillation Complicating Cardiac and Non-Cardiac Surgeries	NCS: mostly thoracic and abdominal surgeries; 3 years after POAF, 1 in 10 patients with stroke; mean CHA2DS2-VASc scores similar in these groups; in more than half the cases, CVA occurred without recurrent AF, so in addition to AF, other vascular risk factors might play a role in causing ischaemic stroke.	^3^
Yoshida	Takuo	2018	Retrospective cohort study	The impact of sustained new-onset atrial fibrillation on mortality and stroke incidence in critically ill patients: A retrospective cohort study	Critically ill patients after non-cardiac surgery in ICU: 9% with POAF; 11 with stroke in 151 patients with POAF (7 with POAF anticoagulated); 5/1567 without POAF (9 on 1567 anticoagulated).	^43^
Butt	Jawad H.	2018	Retrospective cohort study	Risk of Thromboembolism Associated With Atrial Fibrillation Following Non-cardiac Surgery	Similar risk of stroke in patients with POAF or with NVAF (non-valvular AF): 13.0% POAF vs 13.6% NVAF (mean follow-up 3.2 years); lower risk in the group with anticoagulation; patients with POAF, according to this study, could be considered as primary NVAF in terms of risk of thromboembolism (and stroke).	^4^
Higuchi	Satoshi	2018	Prospective cohort study	The study protocol for PREDICT AF RECURRENCE: a PRospEctive cohort stuDy of surveIllanCe for perioperaTive Atrial Fibrillation RECURRENCE in major non-cardiac surgery for malignancy	Patients after surgeries for head and neck, chest or abdomen cancers; strokes both in short- and long-term outcomes; whether AF caused stroke remained unclear.	^17^
Khormaee	Sariah	2018	Retrospective cohort study	Risk of Ischaemic Stroke After Perioperative Atrial Fibrillation in Total Knee and Hip Arthroplasty Patients	Patients undergone to total knee arthroplasty (TKA) and total hip arthroplasty (THA); new-onset POAF (NOAF) more frequently (0.71%) during TKAs compared with THAs (0.66%); of patients with NOAF, 0.08% with stroke within 30 days, 0.1% within 90 days, 0.2% within 120 days, and 0.4% within 365 days; median CHA2DS2-VASC score 4.7 ± 1.2; risk of ischaemic stroke within 1 year after THA or TKA with NOAF 2.7 times higher than without POAF.	^44^
Higuchi	Satoshi	2019	Prospective cohort study	Perioperative Atrial Fibrillation in Non-cardiac Surgeries for Malignancies and One-Year Recurrence	Analysis of POAF recurrence in 1 year follow-up after surgery for malignancy {Higuchi, 2018 #80}; ischaemic stroke in 2 patients (2.6%) with POAF and 3 (0,4%) without POAF (median of the 38th post-operative day); low incidence due to higher prescription of anticoagulation in this study; 1-year AF associated with ischaemic stroke (HR, 18.97), and POAF had similar result (HR, 5.88).	^45^
Lin	Meng-Hsin	2019	Meta-analysis	Perioperative/Post-operative Atrial Fibrillation and Risk of Subsequent Stroke and/or Mortality	About stroke in non-cardiac surgery, they report Gialdini article; higher risk of stroke in patients receiving non-cardiac surgery than cardiac surgery (HR 2.00 vs HR 1.20).	^19^
AlTurki	Ahmed	2020	Meta-analysis	Major Adverse Cardiovascular Events Associated With Post-operative Atrial Fibrillation After Non-cardiac Surgery: A Systematic Review and Meta-Analysis	Median follow-up of 1 month (short-term), POAF associated with an ≈three-fold increase in risk of stroke (2.1% vs 0.7%; OR 2.82); associated with ≈four-fold increase in long-term (from 12 to 28 months) risk of stroke (2.0% vs 0.6%; OR, 4.12). Combining all 18 studies, the overall increase was three-fold.	^20^
Conen	David	2020	Prospective cohort study	Risk of stroke and other adverse outcomes in patients with perioperative atrial fibrillation 1 year after non-cardiac surgery	About 18 000 patients after NCS, 2.2% of them with POAF; incidence of stroke 5.58% patient-years in those with POAF, 1.54% patient-years without POAF; in according to this study, patients with new POAF would benefit from OAC to a similar extent as patients with non-operative AF.	^46^
Koshy	Anoop N.	2020	Meta-analysis	Post-operative Atrial Fibrillation Following Non-cardiac Surgery Increases Risk of Stroke	Stroke in 1.5% of patients with POAF, 0.4% without POAF (medium follow-up of 1.4 ± 1 year); POAF associated with higher risk of stroke (RR 2.51); hazard of stroke lower following thoracic surgery than non-thoracic non-cardiac surgery (RR 1.95 vs RR 3.09).	^47^
Siontis	Konstantinos C.	2020	Retrospective cohort study	Association of New-Onset Atrial Fibrillation After Non-cardiac Surgery With Subsequent Stroke and Transient Ischaemic Attack	550 (13%) patients with new POAF within 30 days after NCS; 452 patients with POAF matched 1:1 with 452 without POAF, median CHA2DS2-VASc score 4 for patients with POAF and 3 with no AF; risk of stroke at 5 years for patients with POAF 10.7%, vs 6.0% without POAF; cumulative incidence of anticoagulant prescriptions at 30 days and 1 year after AF diagnosis 23.6% and 34.6% respectively, but only 9 with anticoagulation at the moment of the stroke.	^28^
Albini	Alessandro	2021	Systematic review and metanalysis	Long-term outcomes of post-operative atrial fibrillation following non-cardiac surgery: A systematic review and metanalysis	POAF associated with four-fold higher long-term risk of stroke (OR 4.05); a single 6-minute episode of subclinical AF sufficient to increase the risk of subsequent stroke {Healey, 2012 #41}, so anticoagulation weakly recommended (by ESC guidelines in 2020), but not high-quality evidence yet.	^21^
Hu	Shi-Min	2021	Letter	Post-operative Atrial Fibrillation After Non-cardiac Surgery and Stroke	Letter to the editor Siontis {Siontis, 2020 #74}, two points analysed: 1) data adjusted for age and CharlsonComorbidityIndex, but (in according to Hu) CHA2DS2-VASc score is more meaningful; 2) another factor to analyse: higher risks of POAF found in patients undergoing emergency surgery compared with those undergoing elective surgery.	^26^
Huynh	Jessica T.	2021	Systematic Review and meta-analysis	Association Between Perioperative Atrial Fibrillation and Long-term Risks of Stroke and Death in Non-cardiac Surgery: Systematic Review and Meta-analysis	More patients with POAF with stroke as compared with those without POAF (1.5% vs 0.9% patient-years; unadjusted RR 2.9); CHADS2 scores from 0 to 4 (predicted annual risk of stroke 2.8–4.0%); CCS: OAC from 1.5% of annual risk of stroke; ACC/AHA: at CHA2DS2-VASc score ≥2 (2.5% of predicted annual risk).	^22^
Koshy	Anoop N.	2021	Retrospective cohort study	Post-operative Atrial Fibrillation and Long-Term Risk of Stroke in Patients Undergoing Liver Transplantation	Patients after liver transplantation (LT); median CHA2DS2VASc score 1 in both groups; patients anticoagulated for AF and at 12-months from LT 9.2% and 18.5% respectively; thromboembolic events (TEE), at a median of 330 days, in 4.5% (almost the 30% at 6 years) of patients (14 strokes, 3 TIAs and 4 systemic embolism); 76.2% of TEE with a CHA2DS2VASc score ≥2; no patients with POAF with OAC had a thromboembolic event; TEE higher in patients with POAF (17.0%) than without POAF (3.1%); so risk of stroke in POAF after LT equivalent to NVAF.	^47^
Semeraro	Gennaro Carmine	2021	Narrative review	Atrial Fibrillation after Lung Cancer Surgery: Prediction, Prevention and Anticoagulation Management	Patients after lung cancer surgery; about stroke, they reported studies by Lin {Lin, 2019 #52}, Koshi {Koshy, 2020 #62}, AlTurki {AlTurki, 2020 #13} and Albini {Albini, 2021 #50}; on anticoagulation, tendency not to prolong AC beyond one month from the restoration of sinus rhythm (for increased risk of bleeding, lower in OAC); ESC 2020 guidelines: long-term OAC recommended (class IIa) in POAF after NCS at risk of stroke.	^18^
Siontis	Konstantinos C.	2021	Letter	Post-operative Atrial Fibrillation After Non-cardiac Surgery and Stroke-Reply	Letter in reply to Hu {Hu, 2021 #64}: 1) CharlsonComorbidityIndex captured multimorbidity, CHA2DS2VASc score only few variables, and results very consistent and comparable using the two scores; 2) they agreed with Hu: emergency non-cardiac surgery more likely to be associated with POAF, but no information on the urgency level of the surgery available in their study.	^27^
Subramani	Yamini	2021	Systematic Review and Meta-Regression Analysis	Incidence, Risk Factors, and Outcomes of Perioperative Atrial Fibrillation following Non-cardiothoracic Surgery: A Systematic Review and Meta-Regression Analysis of Observational Studies	11 studies and 121 517 patients after NCTS; 2 studies (2686 patients with POAF and 106 586 patients with no POAF) reporting data on stroke: POAF significantly associated with post-operative stroke: 0.5% vs 0.1%, OR: 3,95%.	^24^
Siontis	Konstantinos C.	2022	Cohort study	Associations of Atrial Fibrillation After Non-cardiac Surgery With Stroke, Subsequent Arrhythmia, and Death: A Cohort Study	Patients after orthopaedic, gastrointestinal, respiratory, urogenital, neurosurgical, and other surgeries; mean CHA2DS2-VASc score higher in patients with POAF than those with non-operative AF (3.6 ± 1.8 vs 3.3 ± 2.0); incidence of anticoagulant prescriptions at 30 days and 1 year after AF diagnosis 25.1% and 39.1%; 56 patients (out of 550) with POAF with ischaemic stroke or TIA; absolute risk at 5 years 11.0%; rates of ischaemic stroke or TIA similar in the two groups; only 19.6% of patients with POAF with AC at the time of the stroke/TIA, 38.4% in non-operative AF patients.	^29^
Gaudino	Mario	2023	State of the art review	Post-operative atrial fibrillation: from mechanisms to treatment	Concerning stroke, they reported three articles by AlTurki{AlTurki, 2020 #13}, Lin{Lin, 2019 #52} and Siontis {Siontis, 2022 #71}; unclear whether benefit or harm with OAC after POAF.	^2^
Inoue	Keiko	2023	Meta-Analysis	Risk Factors and In-Hospital Outcomes of Perioperative Atrial Fibrillation for Patients with Cancer: A Meta-Analysis	Patients with cancer with POAF with a six-fold increase risk of stroke (OR, 6.37); difficult in AC for risk of bleeding (mainly with cancer).	^23^
Malavasi	Vincenzo Livio	2023	Systematic review and meta-analysis	Atrial fibrillation in vascular surgery: a systematic review and meta-analysis on prevalence, incidence and outcome implications	In vascular surgery: AF before surgery associated with stroke at follow-up (OR 1.61), but not possible to assess outcomes in relation to the different sites of intervention; risk of stroke increased in patients with AF, both in endovascular surgery procedures (OR 1.34) and in open surgery (OR 1.59); incidence of POAF (3,6%) higher than POAF after non-cardiothoracic non-vascular surgery and higher in opensurgical procedure than in endovascular procedures; outcomes of patients with POAF not allow to be assessed, but a higher incidence of stroke/systemic embolism could be expected.	^25^
Tas	Amine	2023	Observational and retrospective study	Perioperative Atrial Fibrillation and One-year Clinical Outcomes in Patients Following Major Emergency Abdominal Surgery	Patients after a major emergency abdominal surgery; at 30 days after discharge, stroke in 1.5% and 0.9% of patients with and without POAF; at 1 year, stroke in 3.4% and 2.7% of patients with and without POAF; median time from discharge until the first stroke 38 days (with POAF); so POAF associated with a similar rate of stroke than patients without POAF (both at 30 days and 1-year follow-up); 89% and 85% of patients with and without POAF with CHA2DS2-VASc score ≥2; patients in OAC after discharge 24%.	^31^
Tas	Amine	2024	Observational and retrospective study	Stroke outcomes in patients with new onset perioperative atrial fibrillation complicating major abdominal surgery compared with patients with new onset non-perioperative atrial fibrillation	Patients with POAF matched 1:5 with patients with new onset non-perioperative atrial fibrillation; similar rates of stroke in the two groups. In patients with POAF, OAC therapy associated with similar rates of stroke compared with no OAC.	^13^
Azimaraghi	Omid	2024	Retrospective cohort study	Role of anticoagulation therapy in modifying stroke risk associated with new-onset atrial fibrillation after non-cardiac surgery	More than 250 000 patients included: POAF associated with increased 1-year ischaemic stroke risk (3.6% vs 2.3%). Risk of ischaemic stroke in POAF mitigated by post-operative OAC: risk 1.81 without OAC, 1.04 with anticoagulation.	^48^

ACC/AHA, American College of Cardiology/American Heart Association; CCS, Canadian Cardiovascular Society; CS, Cardiac Surgery; CVA, Cardiovascular accidents; NCS, Non-cardiac surgery; NCTS, Non-cardiothoracic surgery; NOAF, New-onset atrial fibrillation; NVAF, Non-valvular atrial fibrillation; (O)AC, (Oral) Anticoagulation; POAF, Post-operative atrial fibrillation; RCT, Randomized Controlled Trial; TEE, Thromboembolic events; TIA, Transient ischaemic attack; TS, Thoracic surgery.

### Stroke risk by type of surgery

We analyzed the relationship between the risk of stroke in patients with POAF and the severity of surgery. We categorized the type of surgery (in the articles reporting this information) according to the surgical risk estimate, as reported in the 2022 ESC Guidelines on cardiovascular assessment and management of patients undergoing non-cardiac surgery.^49^ The surgical risk estimate (risk of death at 30 days) ranged from intermediate (1%–5%) to high (>5%) because most studies on POAF analyzed patients after surgeries at a non-low-risk. Stroke incidence in patients with POAF was lower following intermediate-risk compared with high-risk interventions (*Table 2*).

**Table 2:** Risk of stroke related to type of surgery

Surname of last author	Year of publication	Type of surgery	Surgical risk (ESC classification)	Risk of stroke (events per 100 patient-years)-studies with follow-up ≥12 months	Risk of stroke (events per 100 patient-years)-studies with follow-up ≤1 month	Median follow-up (months)	References
Amar	2002	Non-cardiac, thoracic	Intermediate		0.1	<1	^14^
Rao	2012	Esophagectomy	High	0.2		>60	^37^
Gialdini	2014	Non-cardiac (cardiac not analyzed): nervous system, respiratory system, digestive, etc.	Undetermined	1.5		12	^1^
Blackwell	2015	Radical cystectomy (for bladder tumours)	High	24.8		12	^40^
Chaikriangkrai	2015	Lung transplantation	High	1.2		28	^38^
Nassoiy	2016	Gastrectomy for pancreatic, oesophageal or gastric tumours	Intermediate-high	3.2		12	^42^
Ayoub	2018	Non-cardiac (cardiac not analyzed): mostly thoracic and abdominal	Intermediate-high	3.3		36	^3^
Butt	2018	Non-cardiac, non-obstetrical: orthopaedic, ear, nose, throat, arterial vessels, abdominal, etc.	Intermediate-high	4.0		38.4	^4^
Khormaee	2018	Total knee and total hip arthroplasty	Intermediate	0.4	1.0	1 and 12	^44^
Higuchi	2019	Head and neck, chest or abdominal cancers	Intermediate-high	2.6		12	^45^
Conen	2020	Non-cardiac thoracic, non-vascular abdominal, orthopaedic	Intermediate-high	3.7		12	^46^
Siontis	2020	Excluding minor surgery requiring only minimal sedation or local anaesthesia such as: endoscopic ear, nose and throat procedures; endoscopic gastrointestinal, urological, pulmonary procedures; percutaneous (vascular or non-vascular) procedures; and minor skin, dental, ophthalmologic procedures	Undetermined	1.9		60	^28^
Koshy	2021	Liver transplantation	High	3.5		58.8	^47^
Siontis	2022	Orthopaedic, gastrointestinal, respiratory, urogenital, neurosurgical, and other	Intermediate-high	1.6		75.6 ± 58.8	^29^
Tas	2023	Major emergency abdominal: laparotomy (85.2%), laparoscopy (14.8%)	High	3.4	18.0	1 and 12	^31^

### Stroke risk by CHA_2_DS_2_-VASc score

We analyzed the relationship between the baseline risk of stroke in the population and its true subsequent occurrence. The CHA_2_DS_2_-VASc score is a valuable and widely used tool used to predict risk of stroke in patients with atrial fibrillation, including parameters like Congestive Heart Failure, Hypertension, Age ≥75 [Doubled], Diabetes Mellitus, Prior Stroke or Transient Ischaemic Attack [Doubled], Vascular Disease, Age 65–74, and Female gender.^50^ Except for data reported in the study by Koshy *et al*.^47^ all other studies agreed that in patients with POAF the score is higher than in those without AF (as well as than in those with non-peri- or post-operative AF^29^). Tas *et al*.^31^ reported that most patients with POAF had a score ≥2: the score was 2–3 in 48.1% of patients, and >3 in 40.8% of them. Gialdini *et al*.^1^ reported important information on the annual risk of stroke in each class of the CHA_2_DS_2_VASc score: 0.5% with score 0; 0.7% with score 1; 1.0% with score 2; 1.6% with score 3; 2.0% with score 4; 1.9% with score 5; 2.7% with score 6; and 2.9% with score 7 (*Table 3*). These data demonstrate the relationship between the CHA_2_DS_2_-VASc score and the risk of stroke, and provides an opportunity to use this tool to evaluate the need for anticoagulation in patient treatment. In considering the actual risk, these data also provide an opportunity to compare such a risk with that expected in non-POAF patients.

**Table 3:** Risk of stroke in relation with the CHA_2_DS_2_-VASc score in patients with POAF

Surname of first author	Year of publication	Mean CHA_2_DS_2_-VASc score	Risk of stroke related to POAF (events per 100 patient-years)-follow-up ≥12 months	References
Gialdini	2014	variable (1 to 7)	1.5 0.5% score 0, 0.7% score 1, 1.0% score 2, 1.6% score 3, 2.0% score 4, 1.9% score 5, 2.7% score6 and 2.9% score 7	^1^
Blackwell	2015	2.6 ± 1.2 (POAF) vs 1.9 ± 1.3 (no POAF)	24.8	^40^
Chaikriangkrai	2015	CHADS_2_: 1 (both POAF and no POAF)	1.2	^38^
Nassoiy	2016	2.6 ± 1.3 (POAF) vs 2 ± 1.4 (no POAF)	3.2	^42^
Ayoub	2018	2.5 (2–4)	3.3	^3^
Butt	2018	3 ± 1.7 (in both groups, POAF and NVAF)	4.0	^4^
Khormaee	2018	4.7 ± 1.2	0.4	^44^
Conen	2020	CHADS_2:_ 1.9 ± 1.1 (POAF) vs 1.7 ± 1.1 (no POAF)	3.7 - 0 with CHADS_2_ < 2; 8.2 with CHADS_2_ 2–3; 11.6 with CHADS_2_ > 3	^46^
Siontis	2020	4 (POAF), 3 (no POAF)	1.9	^28^
Huynh	2021	CHADS_2_: 0–4; predicted annual risk of stroke of 2.8%–4.0%	1.5	^22^
Koshy	2021	1 (in both groups, POAF and no POAF); 76.2% of TEE with a CHA_2_DS_2_VASc score ≥2	3.5 (TEE, also systemic embolism)	^47^
Siontis	2022	3.6 ± 1.8 (POAF) vs 3.3 ± 2.0 (non-operative AF); 86.73% of POAF patients: score ≥2	1.6	^29^
Tas	2023	3.2 ± 1.4 (POAF): score 0–1 11.1%, 2–3 48.1%, > 3 40.8%; 2.9 ± 1.4 (no POAF)	3.4	^31^

CHADS_2_, Congestive Heart Failure, Hypertension, Age ≥75, Diabetes Mellitus, Prior Stroke or Transient Ischaemic Attack [Doubled]; CHA_2_DS_2_-VASc, Congestive Heart Failure, Hypertension, Age ≥75 [Doubled], Diabetes Mellitus, Prior Stroke or Transient Ischaemic Attack [Doubled], Vascular Disease, Age 65–74, Female; CVA, Cardiovascular accidents; POAF, Post-operative atrial fibrillation; TEE, Thromboembolic events.

### Exploratory data: subgroup and sensitivity analyses

Subgroup (exploratory) analyses were conducted to explore the potential effect. The association between POAF and subsequent stroke remained consistent in both high- and intermediate-risk surgical procedures.

A strong association was observed in studies with older populations (mean/median age ≥70 years).^1,29,31,33,35,37,39-46,48^ Sex-based differences were also explored: while the strength of association varied modestly based on male predominance^30,33-35,38,40,42,47^ in study populations, no clear interaction was evident. It was not possible to perform subgroup analyses based on CHA_2_DS_2_-VASc scores due to a lack of consistent and detailed reporting in the studies included. To test the robustness of our findings, we performed several further sensitivity analyses, e.g. excluding studies with unreported follow-up duration or unclear surgical classification, and excluding studies with prior diagnosis of AF. The pooled odds ratio remained stable (OR 3.00, 95% CI: 1.80–4.99), and heterogeneity remained high (*I*^2^= 90%), suggesting that methodological inconsistencies in this subset of studies are not the only source of the observed heterogeneity (see Supplementary material online, *Figures S1*–S9).

### Anticoagulation use across studies

Among the studies included, 10^3,4,12,16,18,21,28,38,45-47^ provided data regarding anticoagulation use in patients with POAF after non-cardiac surgery. However, the reporting was heterogeneous and often incomplete. Most studies described observational, non-randomized use of oral anticoagulation (OAC), primarily warfarin, with few reporting on NOACs.

In the study by Vallurupalli *et al*.^16^ short-term anticoagulation (30 days) was recommended, with long-term treatment reserved for patients with POAF >48 h and CHA_2_DS_2_-VASc >2. Butt *et al*.^4^ found that OAC use within 30 days was associated with a significantly lower thromboembolic risk (HR 0.52, 95% CI: 0.40–0.67). Other studies^3,28,32^ suggested that patients not anticoagulated at the time of stroke had worse outcomes. Most studies, however, failed to report outcome differences or had too few events to draw conclusions.

Overall, no study directly compared stroke incidence between anticoagulated and non-anticoagulated POAF patients in a controlled manner. The current evidence remains, therefore, inconclusive.

## Discussion

This study underscores the significant risk of stroke associated with POAF, challenging the perception of POAF as a transient and benign condition. The risk of stroke appears substantial, despite being—by indirect comparison—lower than that associated with non-POAF in comparable strata of CHA_2_DS_2_-VASc score. The approximately three-fold increased stroke risk highlights the need for systematic post-operative monitoring and for tailored preventive strategies.

At variance from other studies, our systematic meta-analysis included *any* type of study related to POAF, including trials, reviews of any kind, previous meta-analyses, cohort studies, observational and retrospective studies, as well letters. This allowed access to all peer-reviewed information in the literature to achieve the most comprehensive update on the topic. Our meta-analysis also includes significant recent publications that were not part of prior analyses, such as 4 papers published between 2021 and 2024.^29,31,47,48^ The uniqueness of the present meta-analysis, which stands out from other previously published reports on the topic, primarily stems from the high number of studies (*n* = 19) here included, higher than in all previous similar reports.^20-24,32^ Out of two other systematic reviews and meta-analyses on POAF more recently been published,^51,52^ one of them^52^ primarily analyzed the role of anticoagulation without estimating the incidence of stroke in all patients with POAF or the relation with CHA_2_DS_2_-VASc and the type of intervention, as done in our study. The other review^51^ mainly analyzed the incidence of POAF after non-cardiac interventions without calculating the incidence of stroke or analyzing the role of anticoagulation in this context.

While most previous studies had already reported the association between POAF and stroke, heterogeneity remained a limitation, due to differences in study design, patient populations and surgical types, this latter including total knee arthroplasty and total hip arthroplasty,^44^ radical cystectomy for a bladder tumour,^40^ gastrectomy for a malignancy (pancreatic, oesophageal or gastric),^42^ head and neck, chest, or abdomen cancer surgery,^17^ liver transplantation,^47^ lung cancer surgery,^18^ vascular surgery.^25^ Among various types of surgery, even with similar surgical risk, cancer-related surgeries here appear at particularly high-risk of stroke related to POAF, suggesting an interplay between malignancy, surgery, and POAF in determining the risk of stroke. Patients with cancer, specifically, showed a six-fold increase in stroke risk in the study by Inoue,^23^ a high cumulative incidence of cardiovascular accidents (stroke and myocardial infarction) of 16.7% and 24.8% in the studies by Nassoly^42^ and Blackwell,^40^ respectively. This raises the hypothesis that cancer surgery is an additional risk factor for stroke in patients with POAF, although other studies are needed to confirm this relationship, independent of the immediate surgical risk.

Recent evidence suggests that the burden of AF plays a significant role in determining stroke risk, beyond the mere presence or absence of AF episodes. Patients with POAF often have a lower AF burden, comparable to that observed in AF detected through screening or cardiac implantable electronic devices.^53^ This emerging concept is supported by data indicating a dose–response relationship between AF burden and thromboembolic events.^54^ Although AF burden was not systematically reported in the studies included in our analysis, its potential impact should be considered in future research aimed at refining stroke risk stratification in patients with POAF.

Analyzing the relationship between stroke risk and the CHA_2_DS_2_-VASc score in patients with POAF, similar to what was reported by Gialdini *et al*.^1^ the risk of stroke appears to be lower—in each score class—than risk reported for the same CHA_2_DS_2_-VASc score in non-POAF.^55^ This could be the starting point for re-evaluating the appropriateness of anticoagulation in all such patients. In fact, despite the increased risk of stroke in POAF, indications of how to prevent stroke in such post-surgical patients are not clear. The narrative review by Vallurupalli *et al*.^16^ recommends short-term anticoagulation (30 days) after non-cardiac surgery in patients with POAF. Long-term anticoagulation was suggested only for POAF lasting >48 h and a CHA_2_DS_2_-VASc score >2, always with a shared decision-making process. This is in contrast with recommendations by Conen *et al*.^46^ whereby it was claimed that all patients with newly diagnosed POAF would benefit from OAC to an extent similar to patients with other forms of AF. Albini *et al*.^21^ summarized ‘possible’ indications by weakly recommending anticoagulation, similar to the 2020^56^ and 2024^57^ European Society of Cardiology (ESC) guidelines and to the 2023 American College of Cardiology (ACC)/American Heart Association (AHA) guidelines.^58^ It needs to be acknowledged that there was no high-quality evidence yet to support any such recommendation. The lack of randomized controlled trials and the weak recommendations by guidelines leave, unfortunately, still wide space for indecision. In the studies here analyzed, anticoagulation prescription was usually low in patients with POAF after discharge, but that finding coincides with data showing that few anticoagulated patients had a stroke (none indeed, in the study by Koshy *et al*.^47^).

An important advance has occurred in the report by Azimaraghi *et al*.^48^ demonstrating, in a sample of ∼250 000 patients, that the risk of stroke associated with POAF is mitigated by post-operative anticoagulation. Recommendations need to take into proper account the risk of bleeding resulting from anticoagulation, which is very high in patients having undergone surgery for cancer. This risk, however, is now broadly lower using the non-vitamin K antagonist oral anticoagulants (NOACs) compared with vitamin K antagonists,^59^ an argument that may now tilt the balance towards more anticoagulation in patients with POAF, with an indecision on whether starting already at a projected stroke risk similar to patients with non-POAF, or higher. To this end, Koshy *et al*.^47^ Butt *et al*.^4^ and Tas *et al*.^13^ all underlined that risk of stroke was similar in POAF as in patients with non-POAF in their studies, which is somewhat in contrast with our findings. Even with a greater degree of uncertainty in low-risk classes, we broadly agree with the recommendation of broadly apply anticoagulation in such patients, although recognising that further studies are needed to confirm these findings. Indeed, because of the low stroke risk in patients with device-detected AF (and—by extrapolation—in patients with POAF, which can be considered as a specific example of AF screening done after surgery) and because of the uncertainty surrounding anticoagulation in screening-detected AF, decisions on anticoagulation initiation should be guided by controlled clinical trials.

### Limitations

We recognize limitations in our study. We included all articles reporting on POAF and risk of stroke, without any exclusion criteria; nonetheless our analysis is subject to publication bias because of the possibility that some data were not published or missed in our literature search. Our search was limited to PubMed and did not include other databases (e.g. EMBASE, Cochrane Library) or grey literature, which may have introduced selection or publication bias. Because, however, PubMed lists full papers published in an extended form, we maintain that such selection criteria was the best guarantee of quality of the accessed reports. Second, this is a study-level meta-analysis, without having access to individual patient data, so that we could not group patients according to baseline characteristics. Third, stroke was included as an endpoint, irrespective of the stroke type—ischaemic or haemorrhagic: while this precludes a pure assessment of antithrombotic efficacy, it provides, however, an overall view of treatment efficacy. Fourth, we reported information on the use of anticoagulants only from studies reporting on this, our primary aim being to conclude on the occurrence of stroke. Fifth, we did not distinguish *a priori* between self-limiting (paroxysmal) or persistent POAF, by definition requiring a medical intervention to be converted: recommendations for anticoagulation might be tailored in the future also according to such distinction. Sixth: although for most studies included in the meta-analysis we performed a multivariable regression analysis to correct for confounding variables, we were unable to pool the adjusted risk estimates and outcomes in discrete classes of baseline characteristics and potential prognostic factors, as they were sparsely and variably reported in the primary studies. Seventh, it was not possible in our analysis to assess the impact of the duration of the ECG monitoring on the risk of stroke, predominantly due to inconsistent reporting and heterogeneity across studies. Eight: we recognize that the follow-up duration, limited to 1 year, may not be sufficient to detect long-term outcomes. Ninth: Only a minority of the studies included in our analysis explicitly addressed the presence of preoperative AF. Among these, some studies excluded patients with a prior history of AF, thereby focusing solely on new-onset POAF, while others included such patients and considered prior AF as an independent variable in their analyses, often identifying it as a factor associated with increased stroke risk. No definitive conclusion can be drawn on this issue. Finally, we report a high *I*^2^ value (94%), indicating substantial heterogeneity among included studies, which may be attributed to differences in study design, variability in patient populations, surgery type, surgical techniques, definitions of POAF, follow-up duration, and methods of monitoring post-operative complications.

## Conclusions

Patients with POAF after non-cardiac surgery are at significantly increased stroke risk, albeit to a lesser extent compared with non-POAF at similar CHA_2_DS_2_-VASc score. Structured monitoring protocols during and after hospitalisation are essential, with continuous ECG monitoring recommended at least for high-risk patients, to distinguish between self-limited types of POAF, only occurred because triggered by surgery, and relapsing forms, pointing to a clearly increased individual susceptibility to AF. Future studies should evaluate optimal monitoring durations to distinguish between a single occurrence of AF and relapsing forms; and also assess the clinical relevance of symptomatic vs asymptomatic AF episodes to guide management strategies. For the time being, considering the relatively low-risk imposed on these patients by prolonged NOAC treatment, these agents should be probably the best compromise between efficacy and safety in most such patients.

## Supplementary Material

pvaf056_Supplementary_Data

## Data Availability

All data are incorporated into the article and its online Supplementary Material.
