# Supplementary material for: Post-operative atrial fibrillation and stroke after non-cardiac surgery: a systematic review and meta-analysis
Source: Eur Heart J Cardiovasc Pharmacother. 2025 Sep 30;11(8):682–97. doi: 10.1093/ehjcvp/pvaf056 (PMC12705171; doi:10.1093/ehjcvp/pvaf056)
Supplement: pvaf056_Supplementary_Data [file pvaf056_supplementary_data.docx]

**Postoperative atrial fibrillation and stroke after non-cardiac surgery:**

**a systematic review and meta-analysis**

**Short title: Stroke in Postoperative Atrial Fibrillation**

Jacopo Donati, MD^1^, Doralisa Morrone, MD, PhD^1^, Freek Verheugt, MD^2^

and Raffaele De Caterina, MD, PhD^1^

^1^Chair of Cardiology, University of Pisa and Cardiovascular Division, Pisa University Hospital, Pisa, and

^2^Onze Lieve Vrouwe Gasthuis (OLVG), Amsterdam, the Netherlands

**ONLINE SUPPLEMENT**

Correspondence:

Prof. Raffaele De Caterina

Chair and Postgraduate School of Cardiology-University of Pisa

Cardiology 1 Division-Pisa University Hospital

Via Paradisa, 2 - 56124 Pisa

E-mail: [raffaele.decaterina@unipi.it](mailto:raffaele.decaterina@unipi.it)

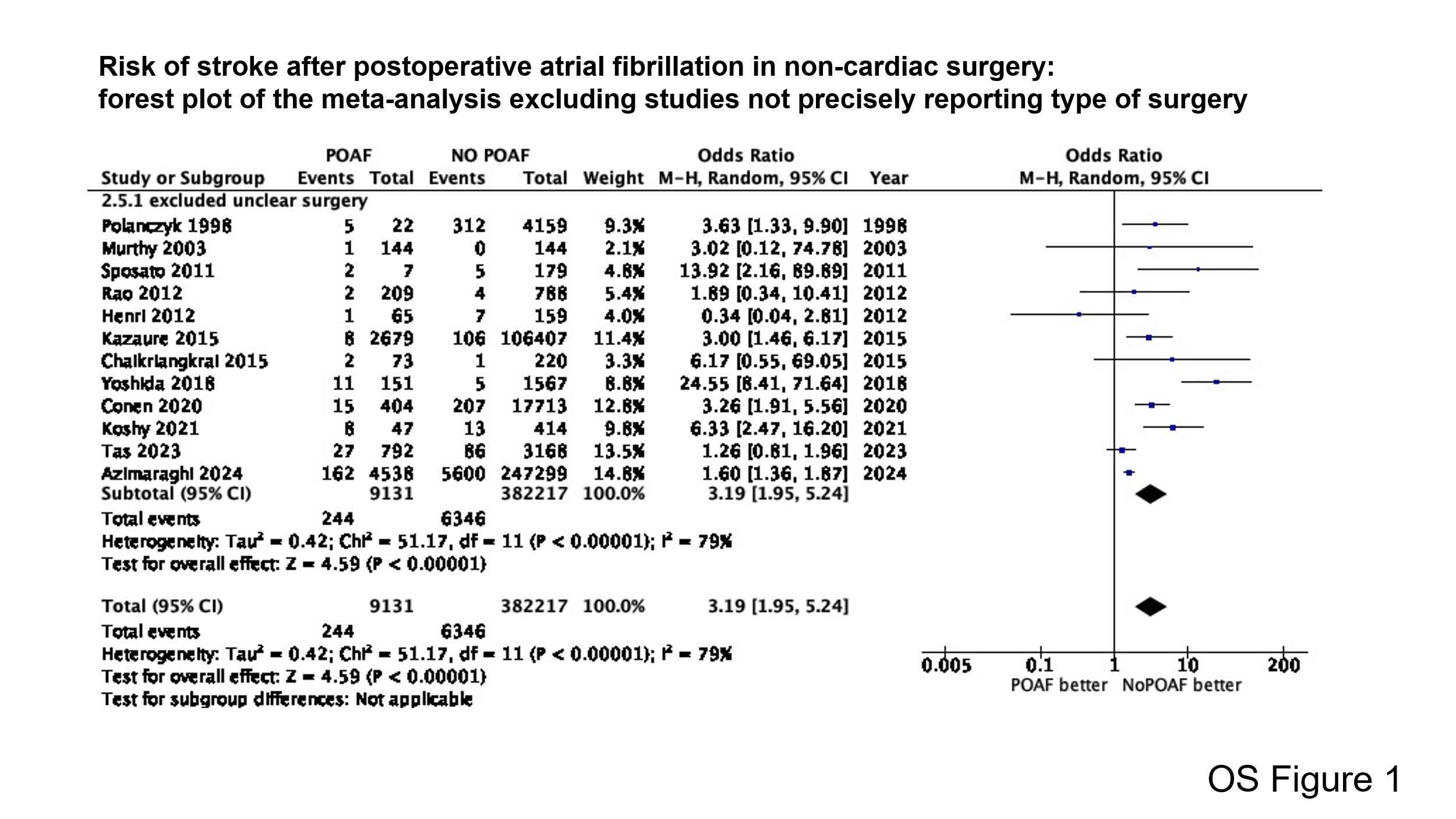


**OS Figure 1: Risk of stroke after postoperative atrial fibrillation in non-cardiac surgery: forest plot of the meta-analysis excluding studies not precisely reporting type of surgery.**

Legend: CI=Confidence Interval; df= degree of freedom; M-H=Mantel-Haenszel; POAF=Postoperative atrial fibrillation.


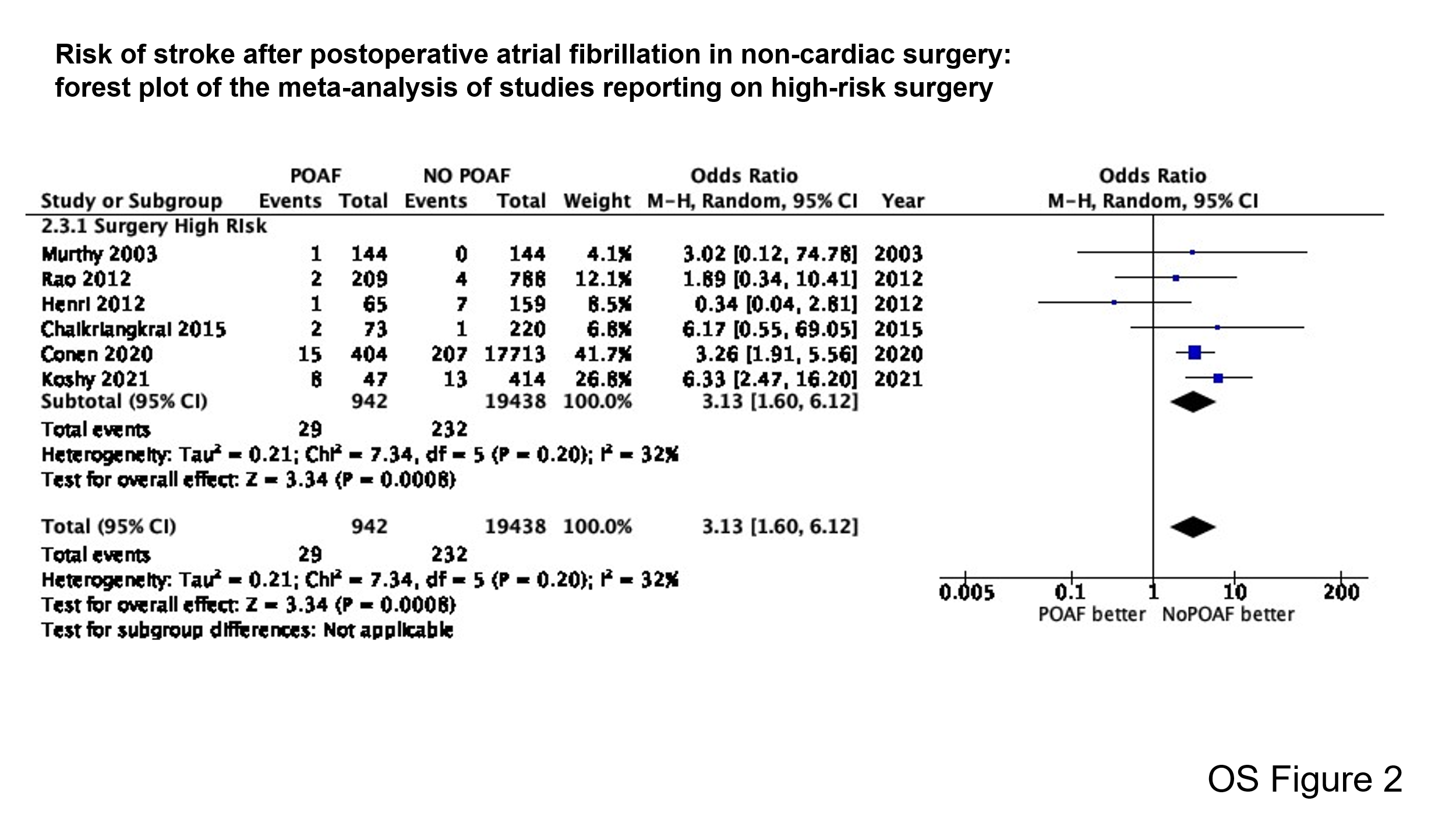


**OS Figure 2: Risk of stroke after postoperative atrial fibrillation in non-cardiac surgery: forest plot of the meta-analysis of studies reporting on high-risk surgery.**

Legend: CI=Confidence Interval; df= degree of freedom; M-H=Mantel-Haenszel; POAF=Postoperative atrial fibrillation.


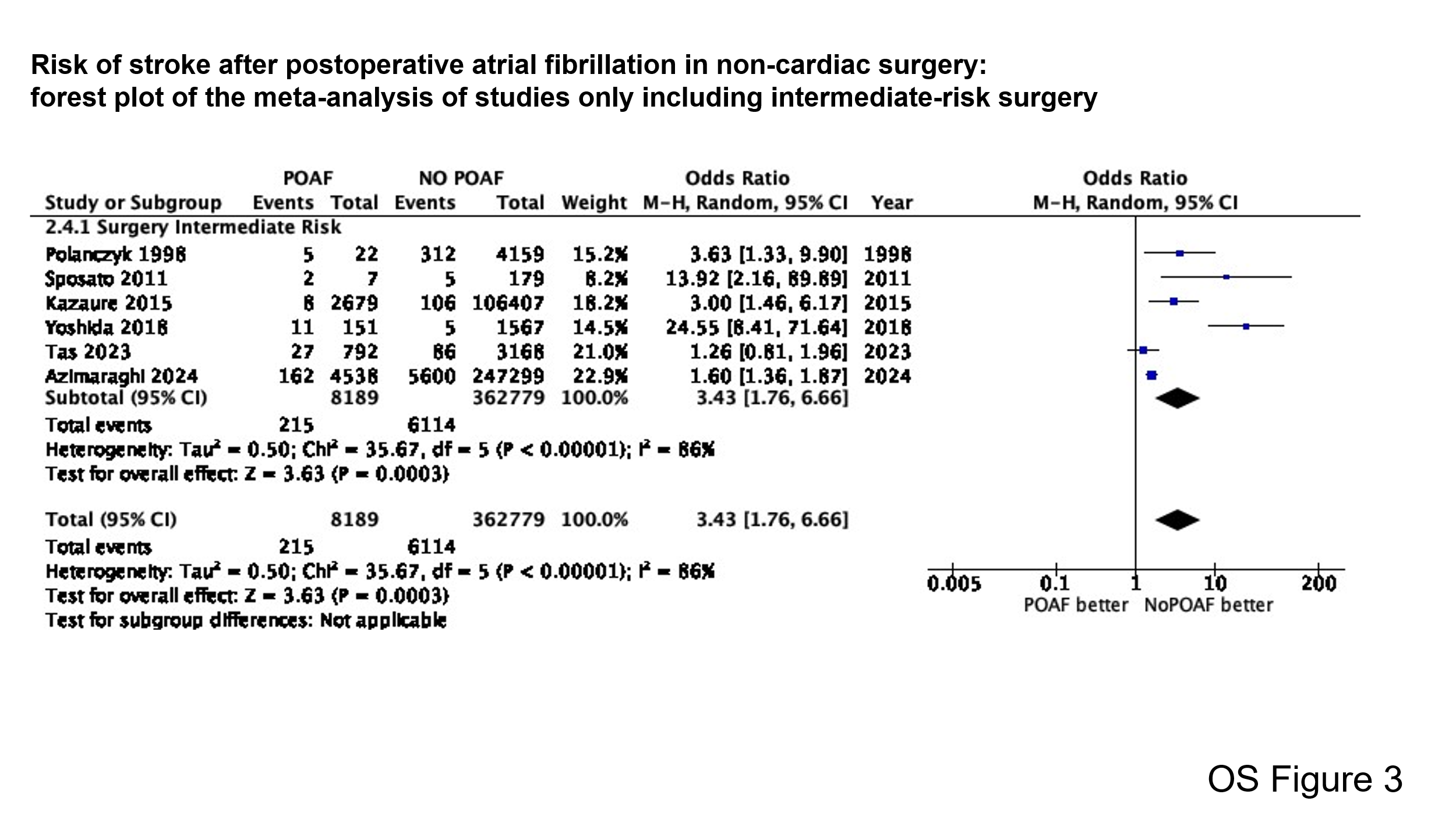


**OS Figure 3: Risk of stroke after postoperative atrial fibrillation in non-cardiac surgery: forest plot of the meta-analysis of studies only including intermediate-risk surgery-**

Legend: CI=Confidence Interval; df= degree of freedom; M-H=Mantel-Haenszel; POAF=Postoperative atrial fibrillation.


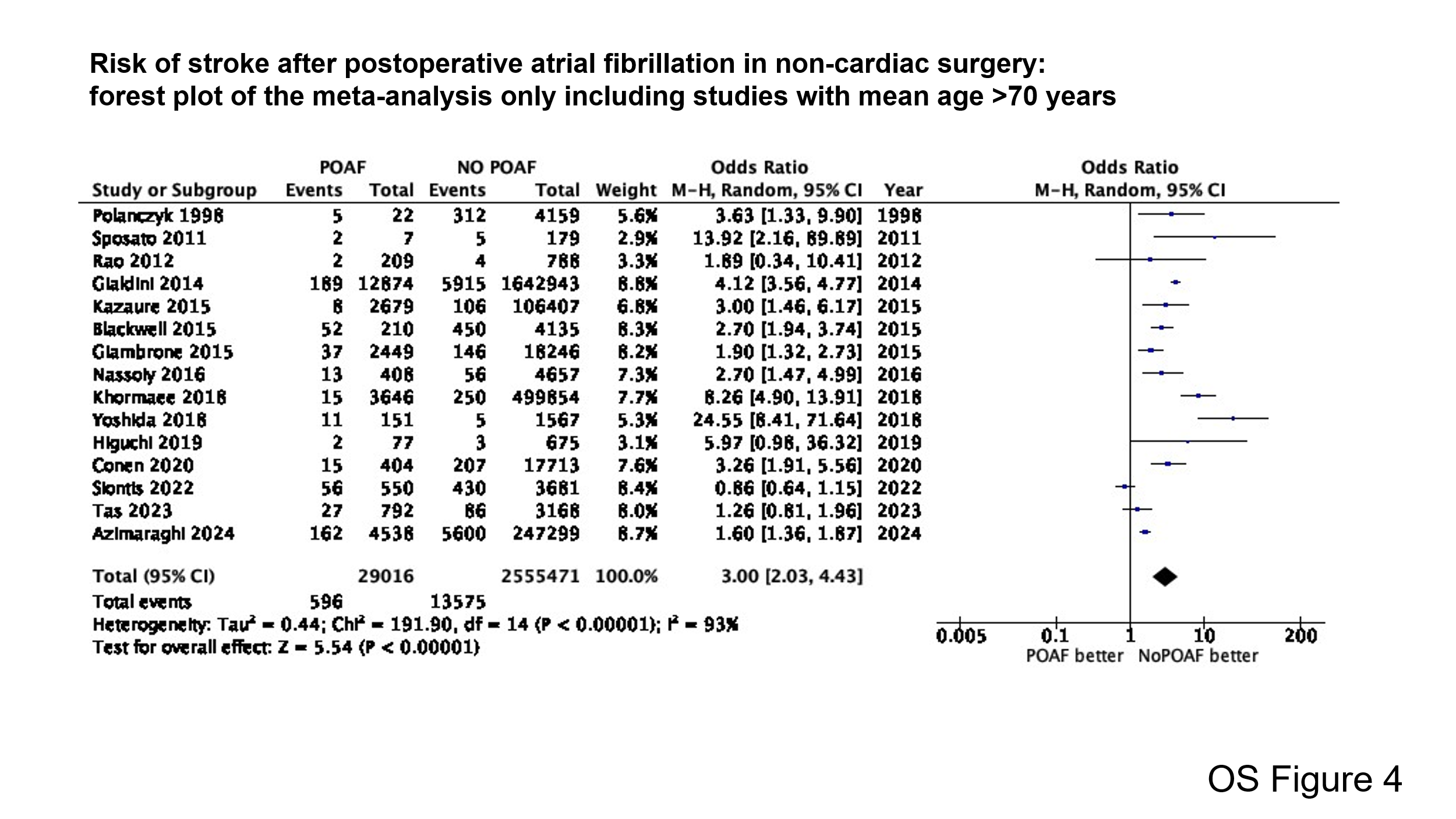


**OS Figure 4: Risk of stroke after postoperative atrial fibrillation in non-cardiac surgery: forest plot of the meta-analysis only including studies with mean age >70 years.**

Legend: CI=Confidence Interval; df= degree of freedom; M-H=Mantel-Haenszel; POAF=Postoperative atrial fibrillation.


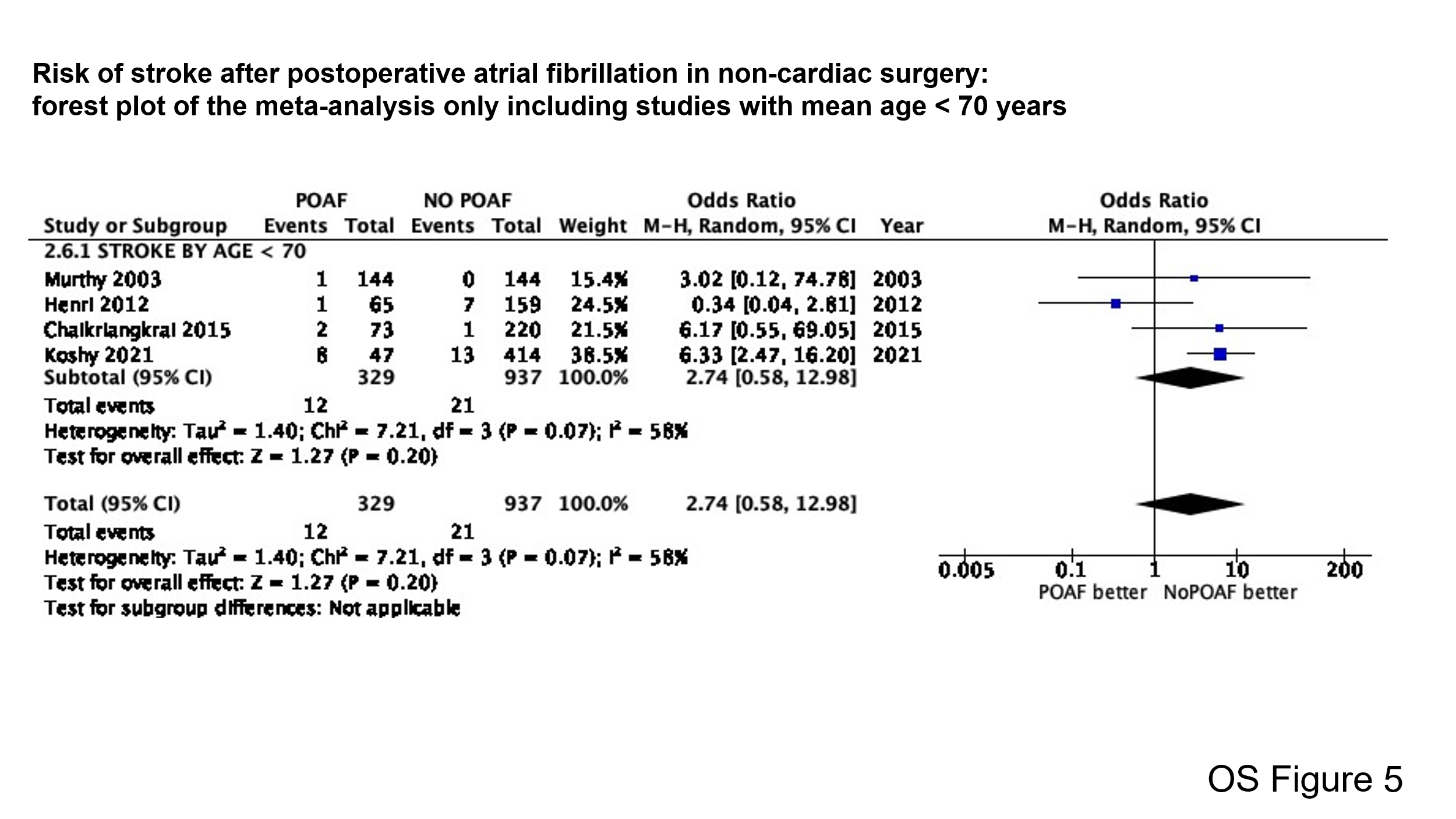


**OS Figure 5: Risk of stroke after postoperative atrial fibrillation in non-cardiac surgery: forest plot of the meta-analysis only including studies with mean age < 70 years-**

Legend: CI=Confidence Interval; df= degree of freedom; M-H=Mantel-Haenszel; POAF=Postoperative atrial fibrillation.

 
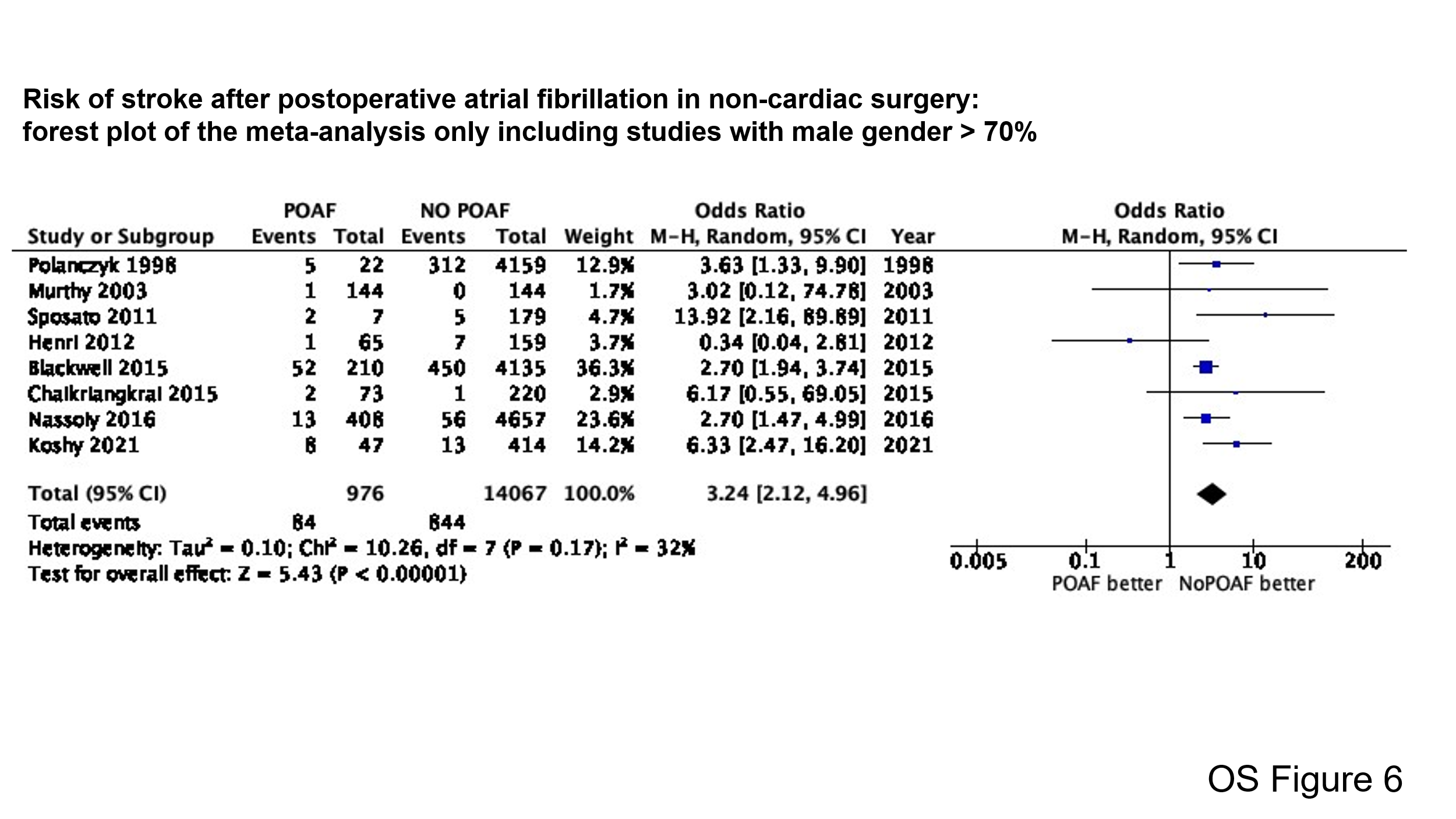


**OS Figure 6: Risk of stroke after postoperative atrial fibrillation in non-cardiac surgery: forest plot of the meta-analysis only including studies with male gender > 70%.**

Legend: CI=Confidence Interval; df= degree of freedom; M-H=Mantel-Haenszel; POAF=Postoperative atrial fibrillation.


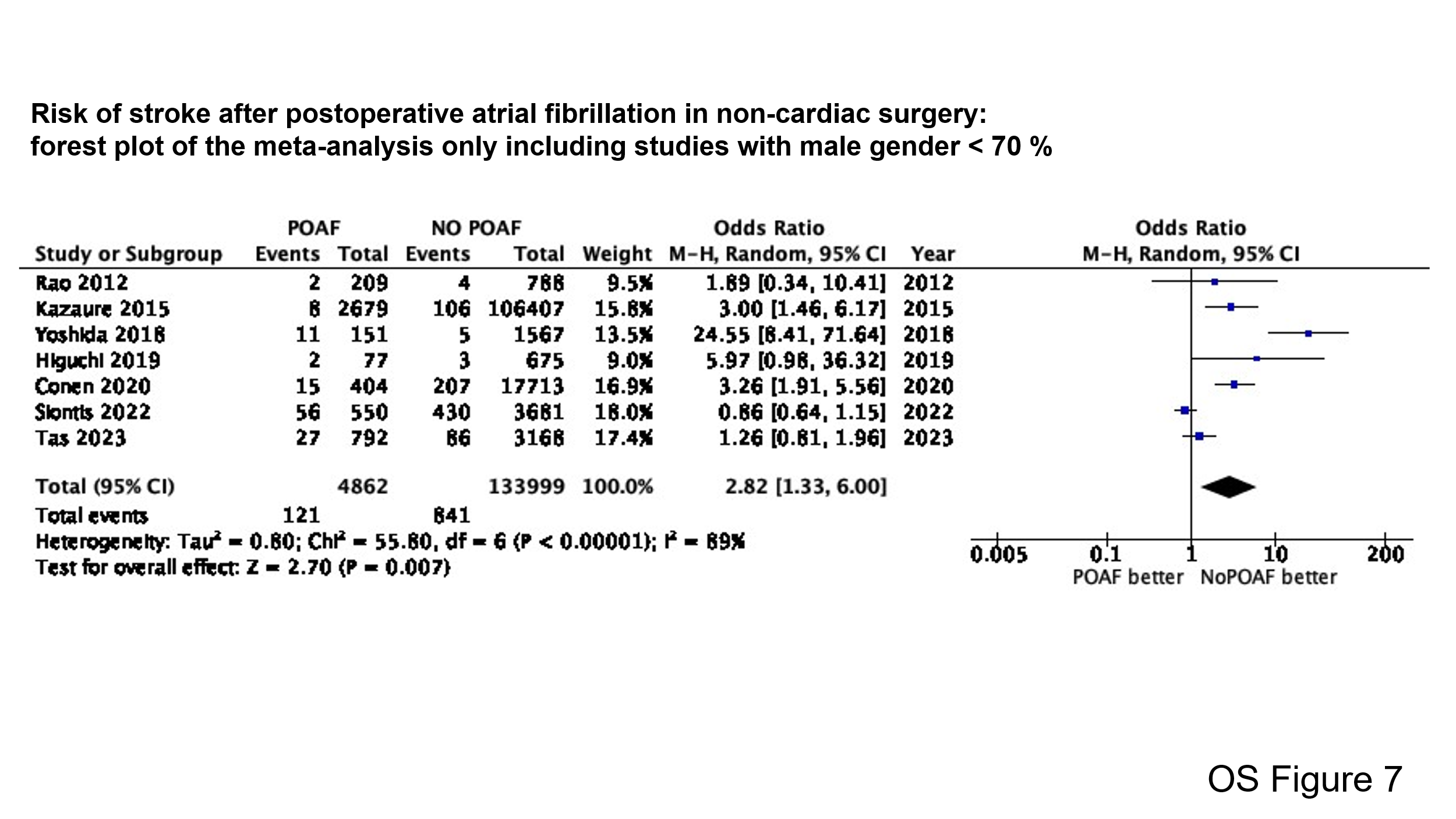


**OS Figure 7: Risk of stroke after postoperative atrial fibrillation in non-cardiac surgery: forest plot of the meta-analysis only including studies with male gender < 70 %.**

Legend: CI=Confidence Interval; df= degree of freedom; M-H=Mantel-Haenszel; POAF=Postoperative atrial fibrillation.


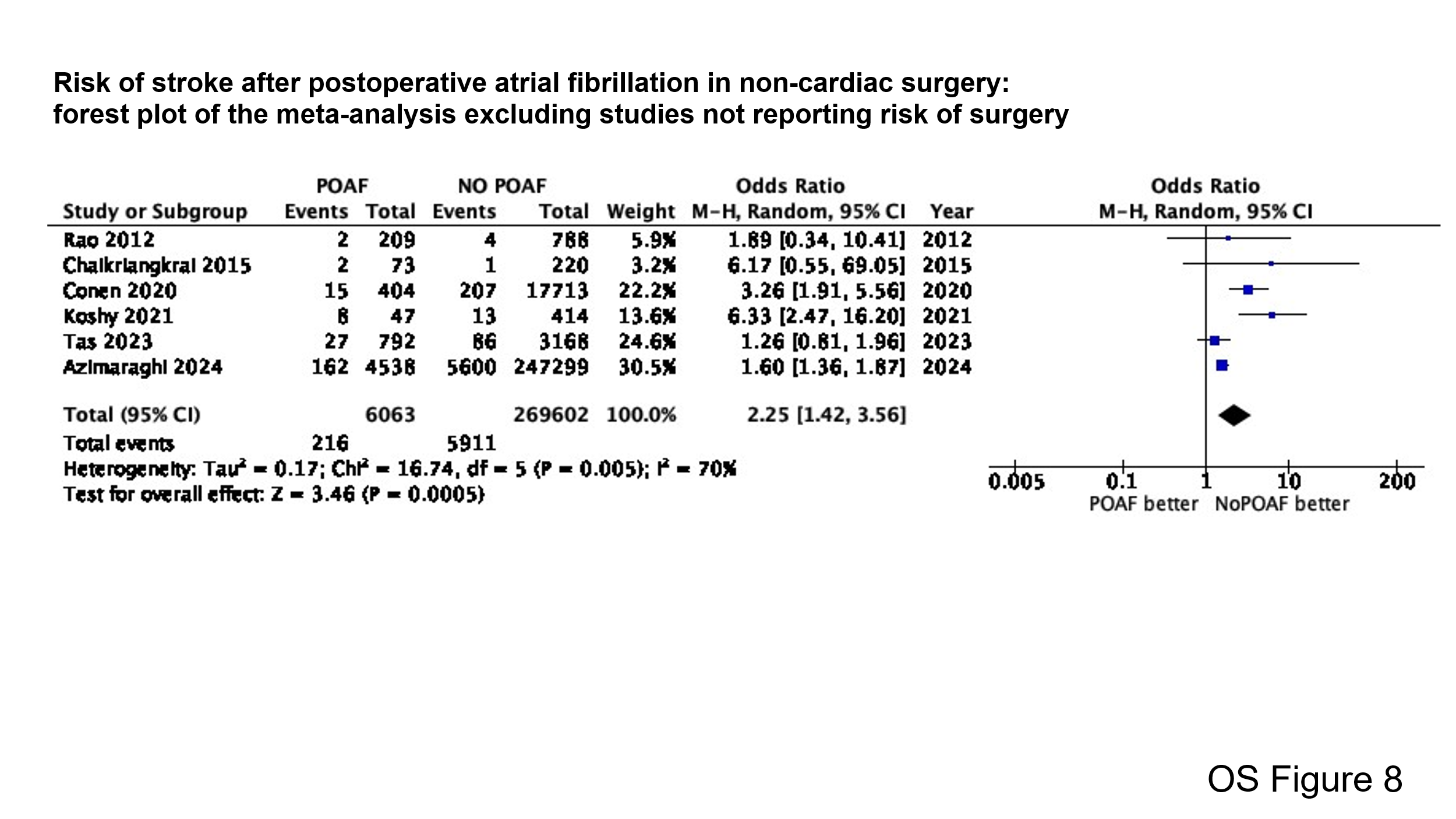


**OS Figure 8: Risk of stroke after postoperative atrial fibrillation in non-cardiac surgery: forest plot of the meta-analysis excluding studies not reporting risk of surgery.**

Legend: CI=Confidence Interval; df= degree of freedom; M-H=Mantel-Haenszel; POAF=Postoperative atrial fibrillation.


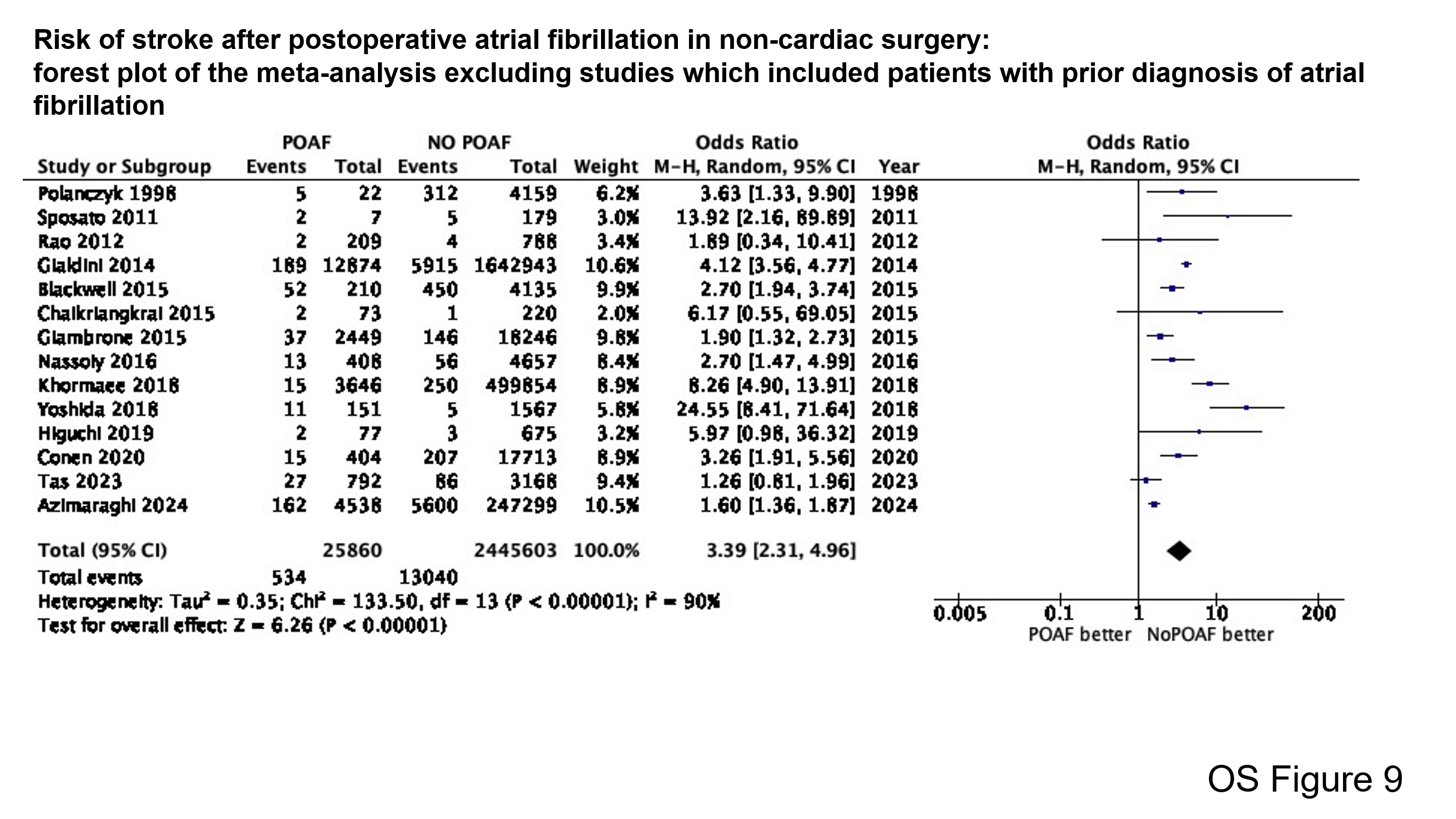


**OS Figure 9: Risk of stroke after postoperative atrial fibrillation in non-cardiac surgery:**

**forest plot of the meta-analysis excluding studies which included patients with prior diagnosis of atrial fibrillation.**

Legend: CI=Confidence Interval; df= degree of freedom; M-H=Mantel-Haenszel; POAF=Postoperative atrial fibrillation.
